# Isolation, molecular identification of lipid-producing *Rhodotorula diobovata*: optimization of lipid accumulation for biodiesel production

**DOI:** 10.1186/s43141-022-00304-9

**Published:** 2022-02-21

**Authors:** Mohamed E. Osman, Asharf Bakery Abdel-Razik, Khaled I. Zaki, Nesma Mamdouh, Heba El-Sayed

**Affiliations:** 1 Botany and Microbiology Department, Faculty of Science, Helwan University, Helwan, Egypt; 2 Genetics Department, Faculty of Agriculture, Ain Shams University, Cairo, Egypt; 3 Plant Pathology Department, Desert Research Center, Mataria, Cairo, Egypt

**Keywords:** Fatty acids, Oleaginous yeasts, *Rhodotorula diobovata*, Molasses, Biodiesel

## Abstract

**Background:**

The increased demand for oil and fats to satisfy the ever-increasing human needs has enhanced the research in this field. Single-cell oils or microbial lipids produced by oleaginous microorganisms are being utilized as an alternative to traditional oil sources. Oleaginous yeasts can accumulate lipids above 20% of their biomass when they are grown under controlled conditions.

**Results:**

In the present study, sixty-five yeasts were isolated from different sources. Using Sudan Black B staining technique, five yeast isolates were selected. Under nitrogen-limited cultivation conditions, the Co1 isolate was the best lipid accumulation potential of 39.79%. Isolate (Co1) was characterized morphologically and identified using the ribosomal DNA internal transcribed spacers regions (rDNA-ITS) from their genomic DNA. The sequence alignment revealed a 99.2% similarity with *Rhodotorula diobovata*. Under the optimized conditions, *Rhodotorula diobovata* accumulated lipids up to 45.85% on a dry biomass basis. *R*. *diobovata*, when grown on different raw materials, accumulated lipid up to 46.68% on sugar beet molasses medium, and the lipid had a high degree of monounsaturated fatty acids which gives biodiesel better quality.

**Conclusions:**

The data suggest that the potent oleaginous yeast, *R*. *diobovata*, together with the use of cheap feedstock raw materials such as sugar beet molasses, can be considered as a promising feedstock for biodiesel production.

## Background

Every year, various kinds of industries expand globally; therefore, energy research has also increased significantly. As a result of the consumption of fossil fuels and their derivatives, several environmental problems and global climate change rapidly appeared [1]. This has created a surge in interest in alternative sources for petroleum-based fuels. An alternative fuel must be technically possible, economically viable, environmentally acceptable, and readily accessible. Therefore it is critical to develop renewable energy sources such as biodiesel [2].

Biodiesel fuels, described as fatty acid methyl esters obtained from various renewable lipid sources, have gained a lot of attention in recent years as an alternative fuel because they are green, biodegradable, renewable, non-food based liquid transportation fuels, safe to the environment, and nontoxic [3, 4]. The use of oleaginous microorganisms such as yeasts, fungi, and microalgae in biodiesel production is a potential solution to overcoming the vital inefficiencies of first-generation biodiesel [5], which competes with human food using vegetable oil as a raw material.

The majority of the lipids in these organisms are triacylglycerols (TAG), which have long chains similar to conventional plant oils [6]. Oil production through microbial cultivation has several advantages, including the ability to produce oils all year long and the absence of the need for large areas of arable land [7]. The oleaginous attribute of yeast renders it an advantage over its competitor, viz. bacteria, molds, and algae, owing to higher rates of proliferation and a proclivity for higher lipid yields [8]. Since yeasts can accumulate up to 70% of their dry weight as lipids [9], they are a potential source of microbial oil [5, 10, 11]. Some oleaginous yeasts have an unusual ability to synthesize unique lipid profiles with high stearic acid percentages and non-negligible palmitic and oleic acid percentages [12]. When one of the nutrients (usually nitrogen) is depleted, lipid accumulation occurs. At the same time, there is an excess of carbon in the medium, such as glucose. When nutrients are depleted, cells do not grow or replicate, but they begin to take up glucose from the medium. Excess sugar is used in lipid biosynthesis. Because the cells no longer grow or divide under nitrogen-limited conditions, the first requirement for the cells is to stop producing energy (i.e., ATP) that is no longer required for the synthesis of macromolecules. During nitrogen limitation, both oleaginous and non-oleaginous yeasts proceed to use carbon, but only oleaginous species metabolize it and increase the ATP/AMP ratio. When the amount of lipid particles increases, the size of these cells enlarges [13, 14]**.** Environmental factors such as temperature, cultivation time, and pH have also been found to influence lipid production and composition [15]. It has long been understood that microbial oil technology can be used under a variety of conditions with a much faster growth rate than plants. Microbes may be genetically engineered and used in closed production processes to manufacture oils from cheap substrates such as molasses, crop residues, wood wastes, or even industrial solid waste [16]. The important parameters of feedstock to produce biodiesel is not only the total quality of fatty acid accumulation during the cultivation, but also the quantity of the fatty acid [17]. The objective of this study was to select wild yeasts isolated from different sources, capable of metabolizing material as a carbon source to obtain better quality intracellular microbial lipids for biodiesel production. Optimization of medium condition and its effect on increasing lipid production was also investigated.

## Methods

### Isolation of oleaginous yeast from rotten fruits, pickles, plant leaves, and juices

One gram from different samples (rotten fruits, pickles, plant leaves, and sugar cane juice) was added to 10 ml of saline solution and subsequently followed by a tenfold serial dilution. An aliquot of 1 ml from each dilution was spread onto plates containing isolation medium which includes (g/L): glucose (20), (NH_4_)_2_SO_4_ (2), KH_2_PO_4_ (0.5), MgSO_4_.7H_2_O (0.2), CaCl_2_.2H_2_O (0.1), and 2% agar in the presence of 3.3 ml of streptomycin solution (10.000 U/ml) [18]. The plates were incubated at 28 °C for 48 h, and then those containing isolated colonies with the morphology typical of yeasts were used for further study. The isolated colonies of yeasts were maintained on yeast extract peptone dextrose (YEPD) slants which includes (g/L): glucose (20), peptone (20), yeast extract (10), and agar 2% [19]. Slants were incubated at 28 °C for 48 h and then stored in a refrigerator at 4 °C until use.

### Isolation of oleaginous yeast from soil samples

Two soil samples were collected from El-Dakhla Oasis, western desert, Egypt, and Wadi El-Natrun, Beheria Governorate, Egypt. A weight of 1 g from each soil sample was added to 50 ml of glycerol enrichment medium which includes (g/L): glycerol 100 ml, (NH_4_)_2_SO_4_ (1), KH_2_PO_4_ (1), MgSO_4_.7H_2_O (0.5), yeast extract (0.2) in a 250 ml Erlenmeyer flask, then incubated at 30 °C for 24 h with shaking at 150 rpm so that the targeted yeasts from the soil would be enriched to a greater number [20]**.** A volume of 1 ml of the above pre-cultured yeasts was added to 9 ml of saline solution (0.9% NaCl) and 10-fold serial dilutions were followed. Then, 0.1 ml of each dilution was spread onto plates contained an isolation medium. The plates were incubated at 28 °C for 48 h. After that, those containing isolated colonies with yeast-like morphology were used for future study. The isolated colonies of yeasts were maintained on YEPD slants incubated at 28 °C for 48 h and then stored in a refrigerator at 4 °C until use.

### Primary screening for oleaginous yeasts

The isolated yeast colonies were screened for their lipid-producing abilities by the qualitative analysis. The technique used for screening was Sudan Black B staining. The isolated yeast colonies were cultivated on a nitrogen-limited medium I which includes (g/L): glucose (50), (NH_4_)_2_SO_4_ (3), KH_2_PO_4_ (0.8), K_2_HPO_4_ (0.2) MgSO_4_.7H_2_O (0.5), and agar 2%. After 5 days of incubation period at 28–30 °C, the cellular lipid content of yeast was analyzed with Sudan Black B staining technique according to the protocol described by Thukar et al. [21]. A thin film of yeast isolate was applied on a clean slide and allowed to dry in the air before heat fixation. The smear was then flooded with Sudan Black B stain, which was left for 15 min until it turned greenish-blue. The remaining stain was then removed, and the slide was thoroughly cleaned with water. Finally, the slide was stained with Safranin for 30 s before being washed, dried, and examined under a microscope. Lipids were stained blue-black or light blue, whereas non-lipid cellular material was pale pink. Yeast isolates with high cellular lipid content were collected and further screened for their lipid production ability.

### Quantitative lipid analysis

The high producer yeast isolates were activated in an inoculation medium, containing (g/L): glucose (40), peptone (5), yeast extract (15) [18]. The inoculum was prepared by transferring one loop full of 24-h yeast culture grown on YEPD agar slant to Erlenmeyer flask containing 50 ml inoculum medium and incubated on a rotary shaker at 150 rpm and 28 °C for 24 h. The cell concentration of inoculum was monitored by counting in a Neubauer chamber until reaching approximately 1 × 10^8^ cells/ml [22]. Then, a volume of 5 ml of inoculum was transferred to 100 ml of the nitrogen-limited medium II which includes (g/L): glucose (100), yeast extract (8), peptone (3). Lipid analysis was done after incubation at 150 rpm and 28 °C for 96 h.

The yeast cells were harvested by centrifugation under 3000 rpm for 15 min. The yeast cells were collected and washed once with distilled water and then dried at 60 °C to constant weight. The biomass was determined gravimetrically. To determine the lipid content in yeast cells, lipids were extracted, dried, and weighed, according to the procedure described by Folch et al. [23]. One gram (dry weight) was extracted with 30 ml of chloroform/methanol (2:1 v/v) at room temperature for 1 h. The solvent mixture containing the extracted lipids was separated from the residual biomass by centrifugation. The extracted lipids in the chloroform phase were separated from the aqueous phase by the addition of 8 ml of saline solution (0.9% NaCl) in a separating funnel with stirring vigorously for phase separation. The upper aqueous phase containing water, methanol, and non-lipid compounds was discarded and the lower phase (chloroform) was filtered using a filter paper containing 1 g of anhydrous sodium sulfate. The chloroform-extracted lipid was collected into weighted measuring glass vials, and the solvent was evaporated. The amount of lipid extracted was determined after drying**.**

Lipid content was determined by the following eq. [24]:

$$\mathrm{SCO}\ \mathrm{productivity}\ \left(\mathrm{Lipid}\ \mathrm{content}\right)=\frac{\mathrm{SCO}\ \mathrm{Weight}\ \left(\mathrm{g}/\mathrm{L}\right)}{\mathrm{Cell}\ \mathrm{dry}\ \mathrm{weight}\ \left(\mathrm{g}/\mathrm{L}\right)}\times 100$$

### Lipid profile analysis using gas chromatography

Extracted lipids were subjected to transesterification reaction by using the methyl esters boron trifluoride method [25]. To obtain fatty acid methyl esters (FAME), simply called biodiesel, an aliquot of the lipid was taken into a capped test tube saponified with sodium hydroxide in methanol. The fatty acids were methylated with boron trifluoride in methanol, extracted with heptane, and determined on a gas chromatograph with FID detector (PE Auto System XL) with the autosampler and Ezchrom integration system. Carrier gas (He); ca. 25 Psi- air 450 ml/min- hydrogen 45 ml – split 100 ml/min.

### Molecular identification of the highest lipid producing yeast isolate

Identification of the most efficient yeast isolate was carried by investigating genotypic characterization, which was performed by sequencing the D1/D2 domains of the gene encoding subunit 26S of ribosomal DNA. The universal primers NL1F (5′GCATATCAATAAGCGGAGGAAAAG3′) and NL4R (5′GGTCCGTGTTTCAAGACGG3′) were used for D1/D2 amplification, according to the methodology described by Kurtzman and Robnett [26]. The genomic DNA of the isolate for the PCR was extracted by the modified method of Harju el al [27].. The PCR product was checked by agarose gel electrophoresis. The amplified product was then purified using Gene JETTM PCR purification kit (Thermo) and sequenced in GATC Company by use ABI 3730xl DNA sequencer by using forward and reverse primers. Only by combining the traditional Sanger technology with the new 454 technology can genomes now be sequenced and analyzed in half the usual project time, with a considerable reduction in the number of coatings and gaps. The nucleotide sequences obtained were analyzed and compared with the sequences deposited in the National Center for Biotechnology Information (NCBI), (http://www.ncbi.nlm.nih.gov/), using the tool Basic Local Alignment Search Tool (BLAST). The phylogenetic tree construction was conducted in MEGA X [28].

### Lipid production using different raw materials by *Rhodotorula diobovata*

The highest producer isolate, *Rhodotorula diobovata*, was cultured in media containing different raw materials (sugar beet molasses, sugar cane molasses, corn steep liquor, and whey) with a concentration of 20%. Five milliliters of activated inoculum was transferred to 100 ml raw material fermentation medium in 250 ml Erlenmeyer flasks. All flasks were incubated on a shaker at 150 rpm and 28 °C for 96 h. Yeast dry biomass, lipid yield, and lipid content were estimated.

### Optimization of culture conditions for biomass production and lipid accumulation by *Rhodotorula diobovata*

The influence of the most suitable sugar beet molasses concentration (5, 10, 15, 20, 25, 30, 35, and 40% v/v), inoculation volume (5, 10, 15, 20, 25, and 30% v/v), temperature (15, 20, 30, 35, and 40 °C), shaking rate (0, 50, 100, 150, 200, and 250 rpm), light presence and absence, pH (3.0, 4.0, 5.0, 6.0, 7.0, 8.0, and 9.0), incubation period (24, 48, 72, 96, 120, 144, 168, and 192 h), additional nitrogen source (peptone, yeast extract, corn steep liquor, urea, casein, beef extract, and tryptone), different concentrations of corn steep liquor (1, 2, 3, 4, 5, 6, and 7%) were tested in a nitrogen-limited media, [29, 30]**.** One parameter was tested at a time. Dry biomass, lipid yield, and lipid content were determined.

### Statistical analysis

All experiments were done in triplicate. One way-ANOVA was performed to calculate significant differences in treatment means. The software Statistica 7.0 by Statsoft was used for the interpretation of the data. Mean separations were performed by Tukey test. A *p* value ≤ of 0.05 was considered significant.

## Results

### Yeast isolation

In this preliminary study, sixty-five colonies with the morphology typical of yeast, colonies with shiny appearance, creamy in texture, and spherical in shape, were isolated from different sources. Twelve isolates from soil samples, as well as ten yeast isolates from rotten fruits (date, plum, peach, grapes, and banana), twenty-one isolates from pickles (cucumber, turnip, olive, onion, carrot, and cauliflower), six isolates from sugarcane juice, and sixteen isolates from the surface of plant leaves (sunflower, grape, maize, soybean, tomato, and cotton leaves) were subsequently used for future study (Table 1).

**Table 1:** Total number of yeast isolates collected from different sources

location	Sample	Number of isolates	Isolate’s code	Location	Sample	Number of isolates	Isolate’s code
Soil samples				**Pickles**			
El-Dakhla Oasis, western desert	The soil around the Clover plant	9	S1:S9	**Local markets**	Olive	6	Ol1:Ol6
					Onion	4	On1:On4
					Carrot	3	C1:C3
					Turnip	3	Tu1:Tu3
Wadi El-Natrun, Beheria Governo-rate		3	S10:S12		Cucumber	3	Cu1:Cu3
					Cauliflower	2	Ca1:Ca2
Rotten fruits				**Plant leaves**			
Local markets	Peach	2	Pe1:Pe2	**Various localities of Egypt**	Grapes	3	Gr1:Gr3
	Grapes	1	G		Sunflower	4	Sf1:Sf4
	Banana	1	B		Maize	2	M1:M2
	Plum	4	Pl1:Pl4		Soybean	3	So1:So3
	Date	2	D1:D2		Tomato	1	To1
Juices					Cotton	3	Co1:Co3
Local markets	Sugar Cane	6	Su1:Su6				

### Screening and characterization of oleaginous yeast colonies using the staining technique with Sudan black B

The lipid accumulation process requires the exhaustion of a nutrient, usually nitrogen, to allow excess carbon to be incorporated into lipids. With the nitrogen-limited growth, the yeast colonies isolated from different sources were screened for their lipid production ability by the qualitative analysis. The technique used for screening was Sudan Black B staining. Based on screening using Sudan Black B staining and examination under microscope, 46 isolates that showed black oil droplets were identified as potential lipid producers (Table 2).

**Table 2:** Microscopic examination of lipid droplets produced by yeast isolates stained with Sudan Black B

Isolate’s code	Lipid detection	Isolate’s code	Lipid detection	Isolate’s code	Lipid detection	Isolate’s code	Lipid detection	Isolate’s code	Lipid detection
S1	++	Pl3	+	On1	+	Ca2	–	M1	+++
S2	+	Pl4	–	On2	++	Su1	+	M2	++
S3	++	D1	–	On3	+	Su2	+	So1	+++
S4	++	D2	++	On4	–	Su3	++	So2	+
S5	–	Pe1	–	C1	+	Su4	–	So3	–
S6	+	Pe2	+	C2	++	Su5	++	To1	+
S7	+	G	++	C3	+	Su6	+	Co1	+++
S8	+	B	+	Tu1	–	Gr1	+	Co2	+
S9	–	Ol1	–	Tu2	++	Gr2	++	Co3	++
S10	–	Ol2	–	Tu3	+	Gr3	+++		
S11	++	Ol3	+++	Cu1	+	Sf1	++		
S12	+	Ol4	–	Cu2	–	Sf2	++		
Pl1	+	Ol5	–	Cu3	–	Sf3	–		
Pl2	–	Ol6	+	Ca1	+	Sf4	++		

Cells containing different amounts of fat droplets were indicated by signs: −: non-lipid-producer, +: low lipid droplets, ++: moderate lipid droplets, +++: high lipid droplets

Some of the strains had multi-lipid bodies. These strains were selected for lipid extraction. Five yeast isolates, Ol3, Gr3, M1, So1, and Co1, accumulated lipid amounts that were greater than 5% of the dry biomass, with the following levels 13.25, 20.83, 24.08, 28.68, and 39.79% of dry biomass, respectively (Table 3). The screening results showed that the yeast isolate Co1 was the highest lipid producer, with lipid content of 39.79%, 1.56 g/L lipid, and 3.92 g/L dry biomass. So, this strain was selected as the potential lipid Producer for further investigation.

**Table 3:** Quantitative analysis of the production of the lipid by the selected oleaginous yeast isolates grown on nitrogen-limited medium II

Isolate’s code	Lipid yield (g/L)^*^	Dry biomass (g/L)^*^	Lipid content (%)^*^
Ol3	1.01^c^	7.62^a^	13.25^e^
Gr3	0.55^d^	2.64^d^	20.83^c^
M1	1.11^b^	4.61^b^	24.08^d^
So1	1.13^b^	3.94^c^	28.68^b^
Co1	1.56^a^	3.92^c^	39.79^a^

*Superscripts a, b, c, d, and e within the same column imply that mean values denoted by the same letters are not significant, Tukey’s test, α = 0.05, *n* = 3

### Identification of the most potent oleaginous yeast isolate

**Fig. 1:**
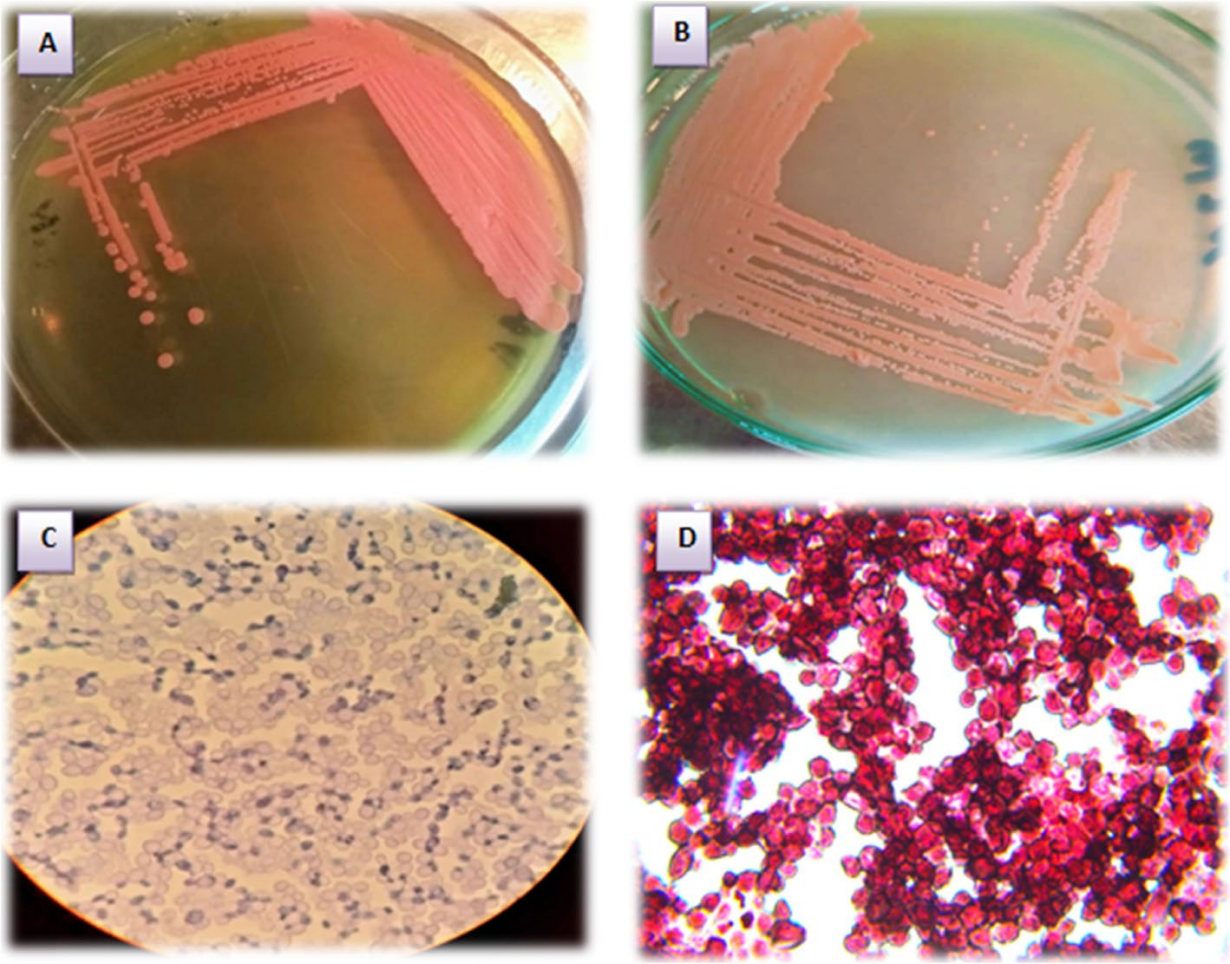
Photo of strain Co1. **A** Co1 strain morphology on YEPD medium. **B** Co1 cells grown on nitrogen-limited medium. **C** Co1 was grown on a nitrogen-limited medium without Sudan Black B staining showing budding observed under the microscope (10 × 100). **D** Co1 was grown on nitrogen-limited with Sudan Black B staining

The results regarding the colony morphology on solid YEPD medium revealed that the Co1 colony appeared as small, orange, circular, smooth, moist colonies with a diameter of 2–3 mm. The yeast cell under the microscope (10 × 100) appeared as ovoidal to circular with multilateral budding (Fig. 1). Sequence analysis of ITS domain and/or D1/D2 domain of the 26S rRNA gene techniques were applied to identify the most potent oleaginous yeast, Co1. Primers used for the amplification of the D1/D2 fragment yielded a fragment of about 595 bp for the Co1 isolate. The nucleotide sequence obtained was aligned to the total nucleotide collection of NCBI using the BLAST tool (http://blast.ncbi.nlm.nih.gov/Blast.cgi). The sequence was deposited into the NCBI database and the acquired accession number was MW714845.

**Fig. 2:**
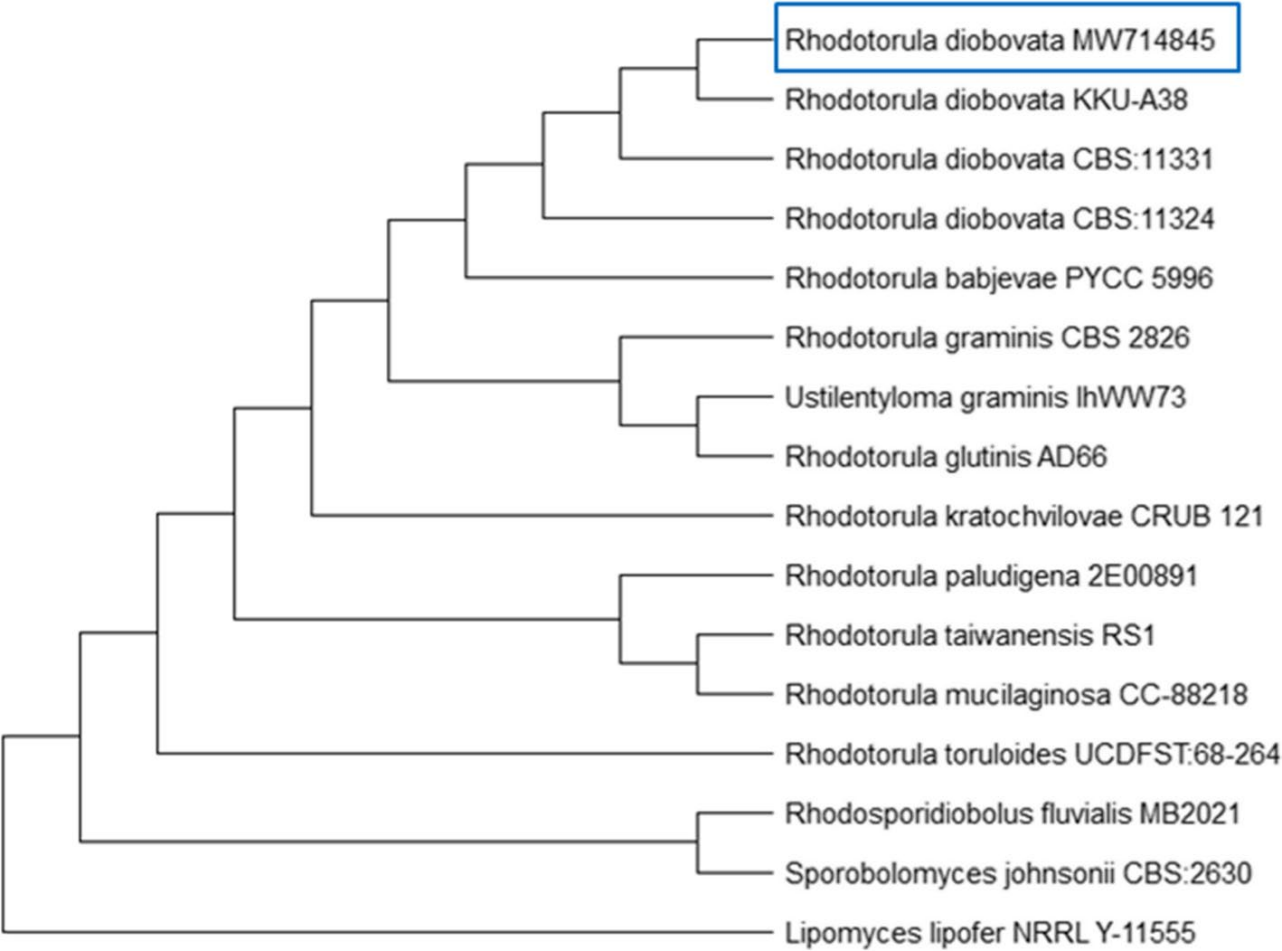
Phylogenetic tree of the D1/D2 domain of the gene encoding subunit 26S of ribosomal DNA sequences of oleaginous yeast strain Co1 with related yeast species in NCBI database

The results showed high D1/D2 sequence similarity (99.8%) with the type strain of *Rhodotorula diobovata*. Therefore, the Co1 strain belonged to *R*. *diobovata*. The phylogenetic placement of this strain with closely related species, based on the D1/D2 sequence is presented in (Fig. 2).

### Effect of raw materials on biomass and lipid production

Four different raw materials “sugar beet molasses, sugar cane molasses, corn steep liquor (CSL), and whey” were evaluated as fermentation media for lipid production in *Rhodotorula diobovata*. As shown in Table 4, sugar beet molasses was revealed to be the most suitable raw material for lipid production, with 4.98 g/L followed by sugar cane molasses with a lipid yield of 4.16 g/L.

**Table 4:** Effect of different raw materials on biomass and lipid accumulation by *Rhodotorula diobovata*

Raw materials	Lipid yield (g/L)*	Dry biomass (g/L)*	Lipid content (%)*
Control	1.56^c^	3.91^d^	39.89 ^c^
Corn steep liquor	0.56^d^	2.05^e^	27.32^d^
Sugar cane molasses	4.16^b^	9.77^b^	42.58^b^
Whey	0.26^e^	4.68^c^	5.56^e^
Sugar beet molasses	4.98^a^	11.43^a^	43.57^a^

*Superscripts a, b, c, d, and e within the same column imply that mean values denoted by the same letters are not significant, Tukey’s test, α = 0.05, *n* = 3

### Optimization of culture conditions for biomass production and lipid accumulation by *Rhodotorula diobovata*

#### Different molasses concentrations

Molasses was used as a carbon and energy source for lipid production of the yeast cells in the current study. To determine the optimum molasses concentrations for maximum biomass production, lipid yield, and lipid content of *R*. *diobovata*, various concentrations of molasses were tested in the nitrogen-limited medium. The maximum lipid yield (4.91 g/L) and lipid content (42.36%) were observed at a molasses concentration of 20%. At molasses concentration of 5%, *Rhodotorula diobovata* showed its minimum lipid yield and lipid content as 1.35 g/L and 17.98% respectively. The maximum biomass production (12.52 g/L) was observed at a molasses concentration of 15%. These results led to the selection of 20% molasses concentration for further optimization.

**Fig. 3:**
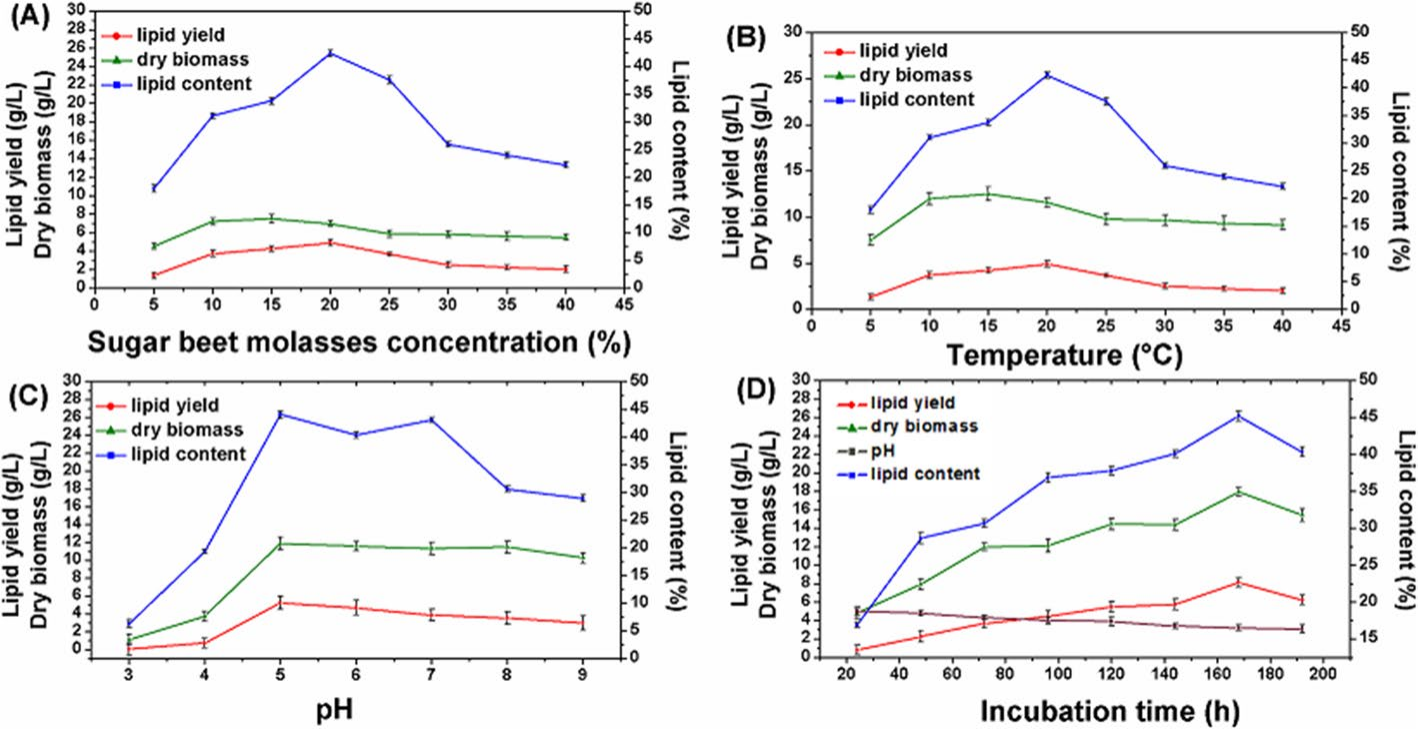
Optimization of culture conditions for biomass and lipid accumulation by *Rhodotorula diobovata*. **A** Effect of different Different molasses concentration on biomass and lipid accumulation by *Rhodotorula diobovata*. **B** Effect of different Different incubation temperatures. on biomass and lipid accumulation by *Rhodotorula diobovata*. **C** Effect of Different pH value on biomass and lipid accumulation by *Rhodotorula diobovata*. **D** Effect of Different incubation time on biomass and lipid accumulation by *Rhodotorula diobovata*. Culture conditions: molasses concentration 20%, initial pH 5.0, temperature 30 °C, and shaking speed 200 rpm. Error bars in figures represent standard deviation means of three biological replicates

The experiments demonstrated that cell biomass and lipid yield, as well as lipid content, were significantly increased by the increase in the molasses concentrations from 5 to 15%. Higher molasses concentrations (20%) caused a decrease in cell biomass but lipid yield and lipid content increased. Molasses concentrations above 20% significantly decreased both lipid yield and lipid content in the yeast (Fig. 3a).

#### Effect of inoculum size

Inoculum size plays a crucial role in yeast single cell oil production. In the present study, the effect of inoculum size on biomass production, lipid yield, and cellular lipid content by *Rhodotorula diobovata* was tested using nitrogen-limited media. Maximum dry biomass (11.84 g/L), lipid yield (5.09 g/L), and cellular lipid content (42.99%) were observed with an inoculum size of 5% v/v, while the lowest biomass (9.54 g/L) was achieved at an inoculum size of 30% v/v (Table 5).

**Table 5:** Effect of inoculum volume on biomass and lipid accumulation by *Rhodotorula diobovata*

Inoculum volume (%)	Lipid yield (g/L)*	Dry biomass (g/L)*	Lipid content (%)*
5	5.09^a^	11.84^a^	42.99^a^
10	4.01^b^	11.17^b^	35.89^b^
15	3.08^c^	10.06^d^	30.62 ^c^
20	2.97^d^	10.27^c^	28.92^d^
25	2.82^e^	10.26^c^	27.49^e^
30	2.14^f^	9.54^e^	22.43^f^

*Superscripts a, b, c, d, e, and f within the same column imply that mean values denoted by the same letters are not significant, Tukey’s test, α = 0.05, *n* = 3

#### Effect of incubation temperature

Incubation temperatures ranging from 15 to 40 °C were checked for the biomass and lipid production in *Rhodotorula diobovata*. The data in Fig. 3b showed clearly that biomass production and lipid yield of *R*. *diobovata* were increased significantly by rising the incubation temperature until reached the maximum values by incubation at 30 °C. At a temperature of 30 °C *R*. *diobovata* produced maximum biomass (11.77 g/L), lipid yield (5.12 g/L), and lipid content (43.51%). By increasing the incubation temperature above 30 °C, the growth started to decline. Considering the present results, the subsequent experiments were performed at the temperature of 30 °C where the maximum lipid content was obtained.

#### Effect of initial pH value of the medium

The influence of the initial pH of the medium on lipid production was studied, using pH values ranging from 3.0 to 9.0. The results represented in Fig. 3c revealed that biomass of *Rhodotorula diobovat*a increased gradually up to pH 5.0 (11.89 g/L) and the same behavior was observed with the produced lipid (5.24 g/L) and cellular lipid content was (44.07%). At acidic pH 3.0 and 4.0, the yeast biomass, lipid yield, and lipid content were decreased. Accordingly, pH 5.0 was selected as the optimum pH for further optimization.

#### Effect of incubation time

In the present study, the effect of the incubation period on biomass production, lipid yield, and lipid content by *R*. *diobovat*a was determined by growing the yeast 24, 48, 72, 96, 120, 144, 168, and 192 h in nitrogen-limited media. The results showed that the biomass and the lipid content increased significantly by increasing the incubation time until reaching the maximum production of biomass (17.95 g/L), the highest lipid yield (8.11 g/L), and lipid content (45.18%) after 168 h (Fig. 3d).

Longer incubation periods resulted in a significant decrease in the biomass yield as well as lipid content. On the other hand, there was a reduction in lipid yield and lipid content between 168 and 192 h.

#### Effect of shaking

The effect of the agitation rate on the yield of cell biomass, lipid production, and lipid content by *R*. *diobovata* under nitrogen-limited conditions was determined by adjusting the agitation speed from 50 to 250 rpm. Maximum biomass of 17.71 g/L, lipid yield of 8.15 g/L, and lipid content of 46.02% were attained by *R*. *diobovata* when the agitation rate was held at 200 rpm. Any further rise in agitation rates above 200 rpm led to a decrease in lipid content (Table 6).

**Table 6:** Effect of shaking rate on biomass and lipid accumulation by *Rhodotorula diobovata*

Shaking rate (rpm)	Lipid yield (g/L)*	Dry biomass (g/L)*	Lipid content (%)*
0	0.87^f^	3.36^f^	25.89^f^
50	3.11^e^	8.01^e^	38.83^e^
100	4.28^d^	10.17^d^	42.08^d^
150	7.91^b^	17.51^b^	45.17^b^
200	8.15^a^	17.71^a^	46.02^a^
250	6.66^c^	15.01^c^	44.37^c^

*Superscripts a, b, c, d, e, and f within the same column imply that mean values denoted by the same letters are not significant, Tukey’s test, α = 0.05, *n* = 3

#### Effect of light

**Fig. 4:**
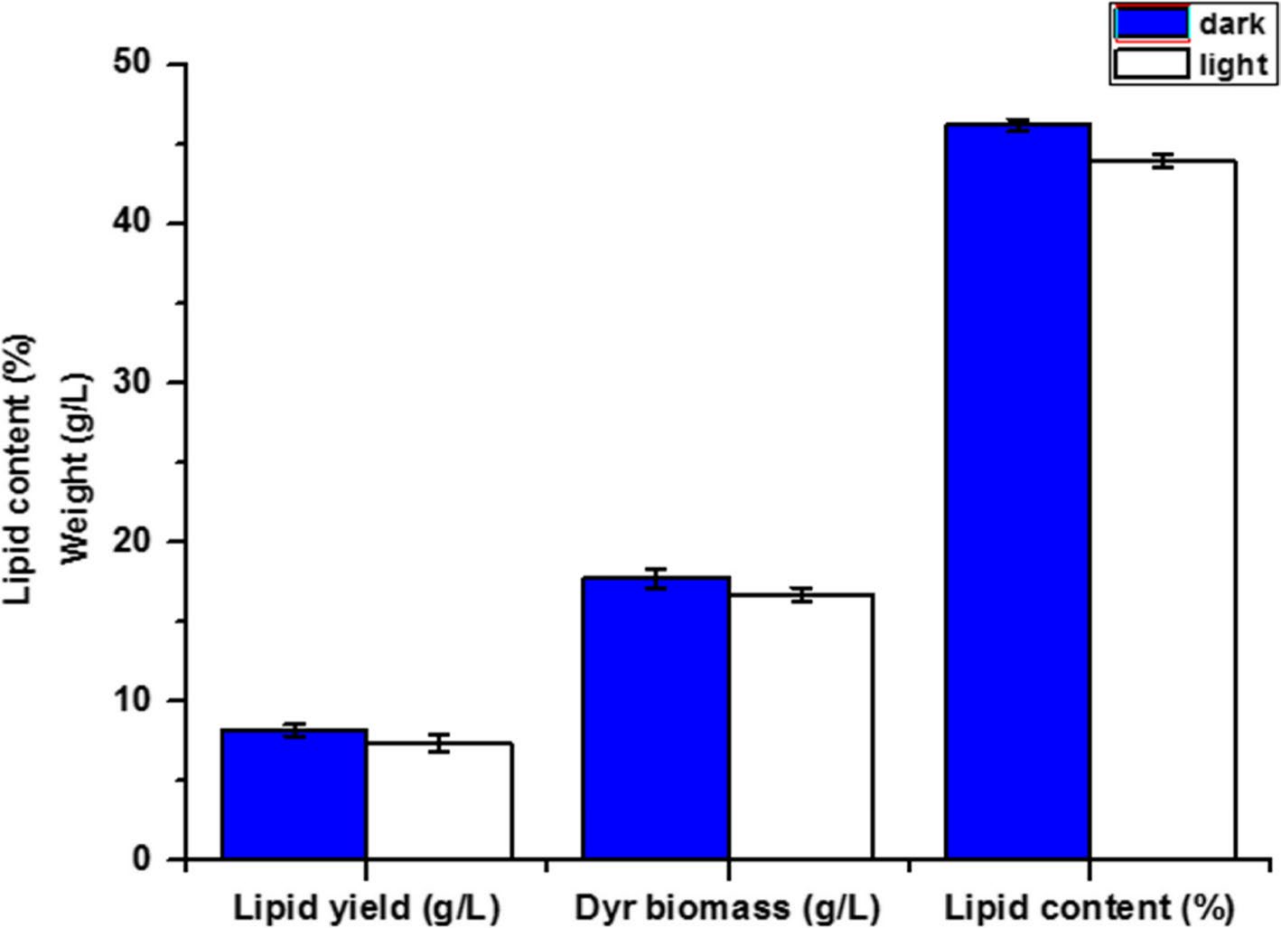
Effect of light on biomass and lipid accumulation by *Rhodotorula diobovata*. Error bars in figures represent standard deviation means of three biological replicates

The effect of light on lipid production was studied; we found that cultivation in light conditions decreased lipid yield but lipid yield was increased in dark conditions (Fig. 4).

#### Effect of organic nitrogen sources

The effect of organic nitrogen such as yeast extract, peptone, corn steep liquor (CSL), urea, casein, beef extract, and tryptone was studied in the molasses medium. Among the tested nitrogen sources, corn steep liquor was proved to be the most suitable nitrogen source for lipid production, with 8.27 g/L of lipid yield and 46.54% lipid content (Table 7). The lowest lipid yield (5.79 g/L) and lipid content (34.86%) were obtained when tryptone was used as a nitrogen source.

**Table 7:** Effect of nitrogen source on biomass and lipid accumulation by *Rhodotorula diobovata*

Nitrogen source (g/l)	Lipid yield (g/L)*	Dry biomass (g/L)*	Lipid content (%)*
Control	8.17^b^	17.65^c^	46.29^b^
Peptone	7.17^d^	17.35^d^	41.33^d^
Yeast extract	6.41^e^	16.83^f^	38.09^f^
Corn steep liquor	8.27^a^	17.77^b^	46.54^a^
Urea	7.33^c^	17.83^a^	41.11 ^e^
Casein	7.13^d^	17.09^e^	41.72 ^c^
Beef extract	6.16^f^	16.15^h^	38.14^f^
Tryptone	5.79^g^	16.61^g^	34.86^g^

*Superscripts a, b, c, d, e, f, g, and h within the same column imply that mean values denoted by the same letters are not significant, Tukey’s test, α = 0.05, *n* = 3

#### Effect of corn steep liquor concentration

**Fig. 5:**
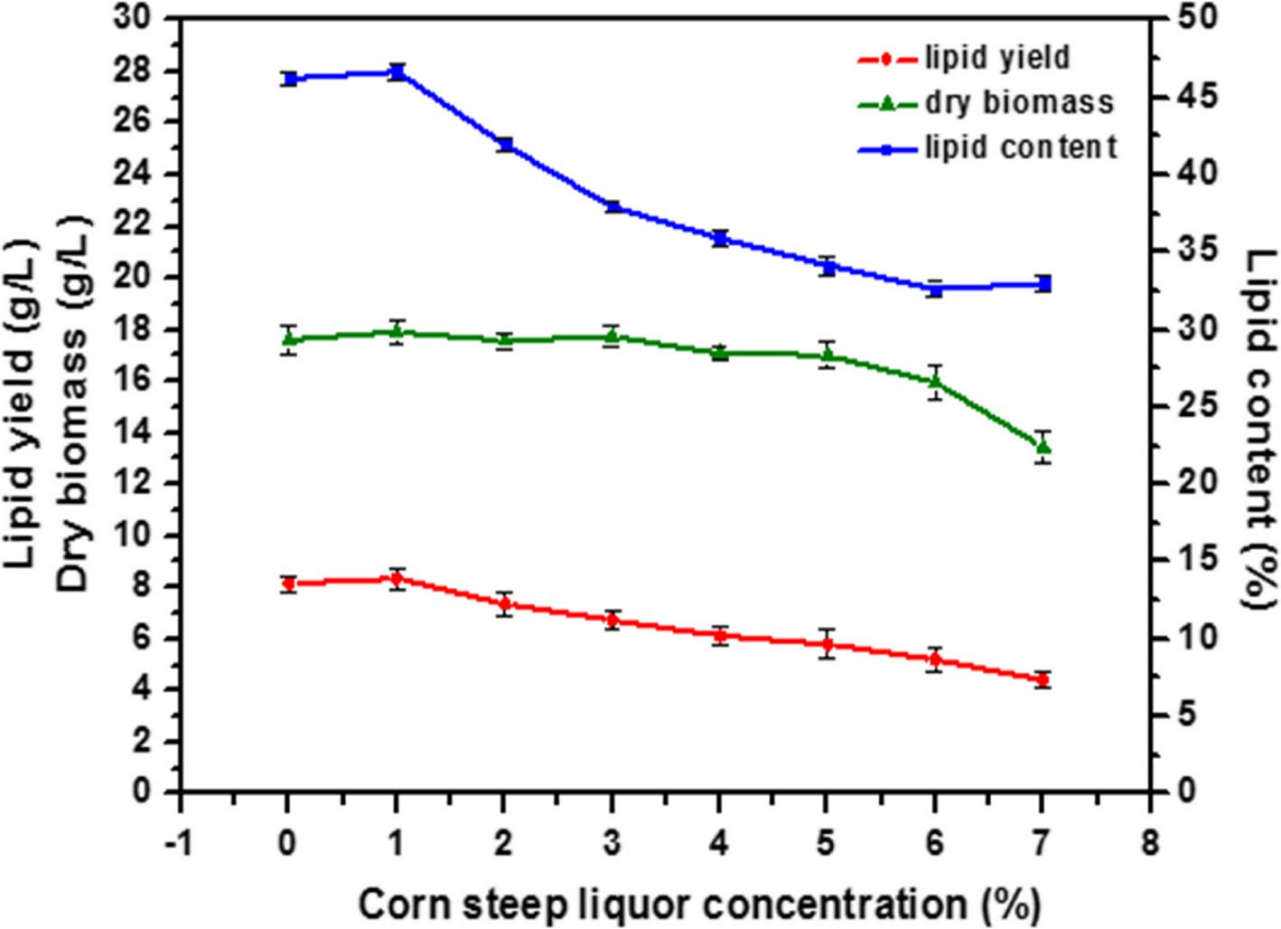
Effect of corn steep liquor concentration on biomass and lipid accumulation by *Rhodotorula diobovata*. Error bars in figures represent standard deviation means of three biological replicates

Effect of corn steep liquor as an additional nitrogen source on lipid production was tested at the different concentrations from 0 to 7%. The maximum lipid yield (8.36 g/L) and lipid content (46.68%) were attained at the CSL concentration of 1%. Applications above 1% resulted in a significant reduction in lipid yield and lipid content (Fig. 5).

### Optimal conditions for biomass and lipid production of *Rhodotorula diobovata*

**Fig. 6:**
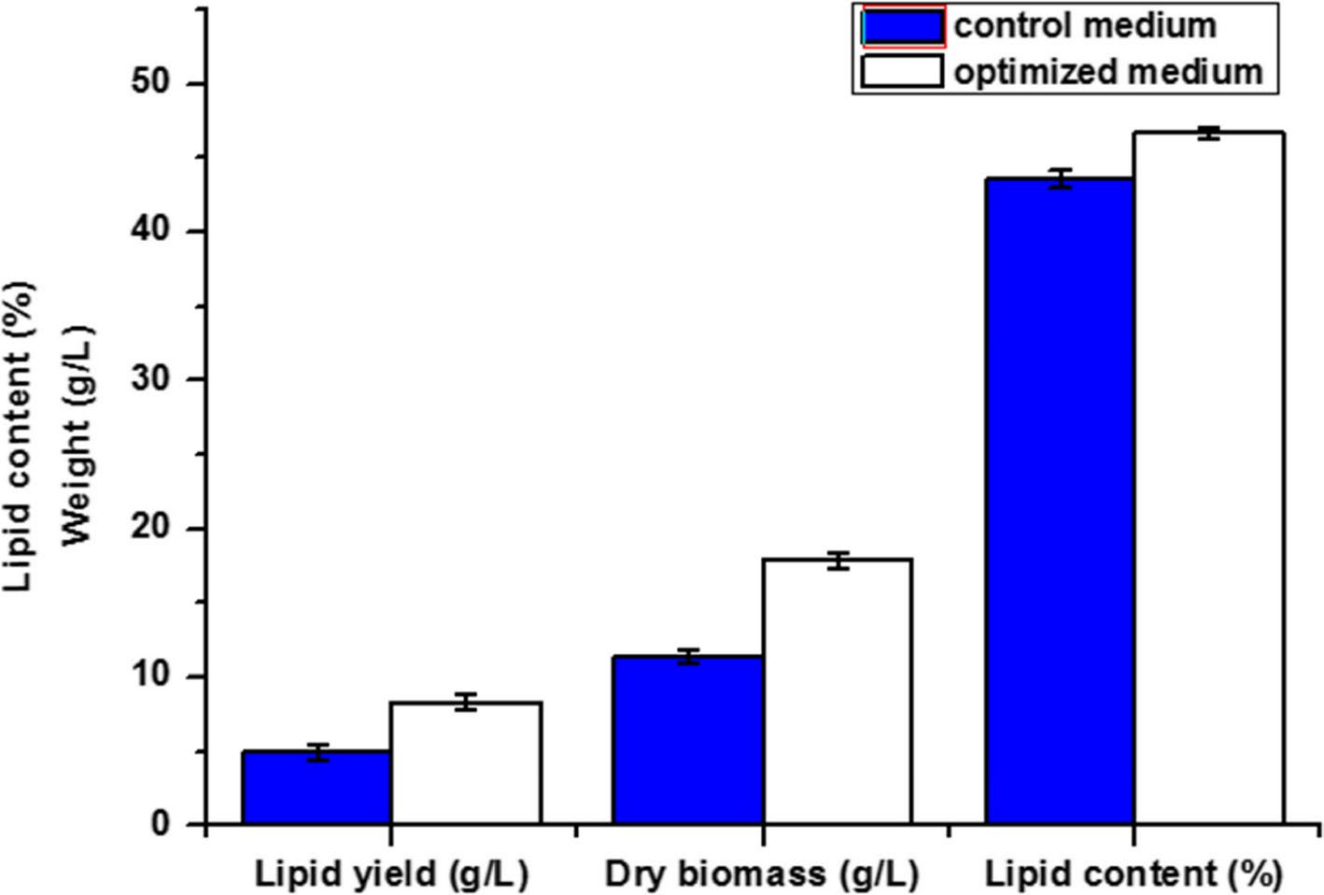
Lipid accumulation potential of *Rhodotorula diobovata* in sugar beet molasses medium under optimized condition Culture conditions. Error bars in figures represent standard deviation means of three biological replicates

According to the previous studies which were carried out in the present investigation, *Rhodotorula diobovata* is a high lipid-producing yeast with the potential of industrial applications. Its lipid yield reached 4.98 g/L with a lipid content of 43.57% when cultivated on sugar beet molasses before optimization, but the lipid yield reached up to 8.36 g/L and lipid content increased to 46.68% when cultivated on sugar beet molasses under optimized conditions (Fig. 6). The optimal fermentation conditions were also developed as 50 ml of medium with molasses concentration 20% and corn steep liquor 1%, (pH 5.0) in a 250-ml Erlenmeyer flask with 5% inoculum under orbital shaking at 200 rpm for 168 h at 30 °C.

### Fatty acid profile

The FAME lipid profiles of *R*. *diobovata* were assessed in nitrogen-limited medium and optimized molasses medium, with results shown in Table 8. The major fatty acids in *R*. *diobovata* were oleic (C18:1), palmitic (C 16:0), and stearic (C 18:0) acids. Minor fatty acids were linoleic (C18:2), and lignoceric (C24:0) acids on both nitrogen-limited and molasses media. Other fatty acids were present in trace amounts (data presented in Table 8). Caprylic acid (C8:0), Undecanoic acid (C11:0), and Lauric acid (C12:0) were not detected in the fatty acid profile of *R. diobovata* cultivated in molasses medium. The Fatty acid composition of the lipid fraction of the yeast *Rhodotorula diobovata* cultivated in the nitrogen-limited medium under optimal conditions consisted of 25.15% saturated fatty acids. The content of monounsaturated was 69.66%, highlighting C18:1, which accounted for 66.58%, and the content of polyunsaturated represented 5.25%. For cultivation in treated sugar beet molasses, the proportion of saturated fatty acids was 35.74%, 60.5% monounsaturated highlighting C18:1, which accounted for 59.37% and 3.75% polyunsaturated.

**Table 8:** Fatty acids profile of lipids accumulated by *R. diobovata* on nitrogen-limited medium and molasses medium using gas chromatography

Type of fatty acids	Fatty acid profile	Relative distribution in nitrogen-limited medium	Relative distribution in molasses medium
Saturated fatty acids	Caprylic acid (C8:0)	3.35%	nd
	Undecanoic acid (C11:0)	0.14%	nd
	Lauric acid (C12:0)	0.57%	nd
	Myristic acid (C14:0)	0.84%	0.86%
	Palmitic acid (C16:0)	14.46%	18.00%
	Heptadecanoic acid (C17:0)	0.19%	1.04%
	Stearic acid (C18:0)	3.54%	12.59%
	Arachidic acid (C20:0)	0.23%	0.50%
	Behenic acid (C22:0)	0.46%	1.00%
	Lingnoceric acid (C24:0)	1.37%	1.75%
	**Total saturated fatty acids**	**25.15%**	**35.74%**
Unsaturated fatty acids	Palmitoleic acid (C16:1)	2.72%	0.92%
	Hexagonic acid (C16:3)	1.08%	1.17%
	Oleic acid (C18:1)	66.58%	59.37%
	Linoleic acid (C18:2)	3.83%	2.36%
	Linolenic acid (C18:3)	0.34%	0.22%
	Gondoic acid (C20:1)	0.36%	0.21%
	**Total unsaturated fatty acids**	**74.91%**	**64.24%**

nd: not detected

## Discussion

Generally, oleaginous yeasts are selected when it comes to lipid production. Oleaginous yeasts are well-studied microorganisms and include members of the genera *Candida*, *Rhodosporidium*, *Yarrowia*, *Cryptococcus*, *Rhodotorula*, *Lipomyces*, and *Trichosporon*, which can sometimes accumulate lipids up to 80% w/w of their dry cell weight [31, 32]**.** Yeasts grow commonly in moist environments where there is an abundant supply of sugar-rich sources. They are widely present with leaf, fruit surfaces, and roots as well as various types of food. Yeasts are present in the atmosphere as well as certain habitats such as agricultural and forest soil, freshwaters, and marine waters from the ocean surface to the deep sea [33].

Yeast isolated from different sources were screened for their lipid production ability using Sudan Black B staining. The yeast lipid bodies were obvious as black droplets inside the oleaginous yeast cells under the optical microscope. While this technique did not allow detailed insight into the cellular lipid content of the yeasts, it did provide, at least partially, information on the lipid accumulation capacity of the tested yeast isolates [34]. Five yeast isolates were selected and further screened for lipid extraction. The screening results showed that the yeast isolate Co1 was the highest lipid producer with a lipid content of 39.79%. Mukhtar et al. [35] reported that *Yarrowia lipolytica* produced a maximum amount of lipids of 22.8%. Pan et al. [34] analyzed few strains of yeasts like *Rhodosporidium toruloides* and *Rhodotorula glutinis* that produced 38.94% and 23.71% oils in xylose containing medium. Similarly, some other strains of yeasts like *Candida utilis* and *Candida tropicalis* showed lipids percentages of 23.44% and 24.88%, respectively. So, this strain was selected as the potential lipid producer for further investigation.

The morphological characteristics of this yeast isolate matched those of *Rhodotorula sp.* described by Kutty and Philip [33]. Al-Turki et al. [36] also reported the multilateral budding and the absence of both ascospores and pseudohyphae in *Rhodotorula sp*. Based on the colony and cell morphology of the yeast strain, it was suggested that Co1 belongs to the genus *Rhodotorula.* Identification of the potential oleaginous yeast Co1 has been confirmed using molecular studies. Sequence analysis of D1/D2 domain of 26S rRNA gene confirmed that Co1 strain belonged to *Rhodotorula diobovata*. Pan et al. [34] identified 20 oleaginous yeasts by sequencing the D1/D2 domains of 26S rDNA (rRNA gene). Mohamed et al. [37] also used ITS to identify three isolates of *R*. *mucilaginosa* and three of *R*. *glutinis*.

Four different raw materials were evaluated as fermentation media for lipid production in *Rhodotorula diobovata*. Sugar beet molasses showed to be the most suitable raw material for lipid production. Under specific nutrient-deficient circumstances, oleaginous microorganisms produce lipids. Molasses is a suitable substrate for microbial lipids because of its high carbon (mainly sucrose) and low nitrogen content, but its use necessitates the addition of additional nitrogen sources [38, 39]. Furthermore, sugar beet molasses provides a low-cost and readily available biodiesel substrate. As a result, in the current work, it was employed as a substrate to design and improve an industrial medium for biodiesel synthesis.

The use of molasses as a substrate was reported for oleaginous yeasts such as *Rhodotorula kratochvilovae* (syn, *Rhodosporidium kratochvilovae*) SY89 [40], *R. glutinis* [41], *Candida lipolytica*, *C. tropicalis*, and *Rhodotorula mucilaginosa* [2], *Geotrichum* (*syn*, *Trichosporon*) *fermentans* [4], R. glutinis *CCT 2182*, *Rhodotorula* (*syn*, *Rhodosporidium*) *toruloides CCT 0783*, *R.* *minuta* *CCT1751*, and *Lipomyces starkeyi DSM 70296* [38] for the production of biomass and hence lipid yield.

Different concentrations of molasses were tested in the nitrogen-limited medium. The molasses concentration of 20% revealed the maximum lipid yield (4.91 g/L) and lipid content (42.36%). Kongruang et al. [17] reported that molasses was a good industrial carbon source and the maximum cellular lipid content was attained by *R*. *toruloides TISTR 5154* (32.35%) followed by *Yarrowia lipotica* 5054. (21.51%), *Y*. *lipotica 5151* (21.09%). *R*. *toruloide TISTR 5149* had the lowest lipid content 10.24%.

When inoculum size increased above 5%, the lipid yield was significantly decreased. This result corresponds with the findings of Chen et al. [42] who stated that inoculum size of 5% v/v was the optimum value for maximum biomass and lipid production by *Cutaneotrichosporon cutaneum* (*syn*, *Trichosporon cutaneum*) grown on corncob acid hydrolysate. The previous studies have reported that the optimum inoculum size of 10% v/v was also optimum for higher biomass and lipid yield as well as lipid content by *Phenoliferia glacialis* (*syn*, *Rhodotorula glacialis*) DBVPG4875 and *Rhodotorula kratochvilovae* (*syn*, *Rhodosporidium kratochvilovae*) *SY89* [39]. The large inoculum size may cause less biomass production due to the limited availability of medium components for yeast to give maximum cell biomass growth [35].

Cellular lipid accumulation is affected by temperature. Temperature affects microbial growth rate, lipid synthesis and alters cellular fatty acids composition [43]. At a temperature of 30 °C, *R*. *diobovata* produced maximum biomass (11.77 g/L), lipid yield (5.12 g/L), and lipid content (43.51%). These results at 30 °C are in agreement with the results of other authors [44].

Studying the influence of the initial pH of the medium on lipid production has revealed that the maximum lipid yield was observed at pH 5.0. The optimum pH for biomass and lipid production for *R*. *toruloides* DMAKU3-TK16 [45], *Rhodotorula kratochvilovae* [39], *Rhodotorula glutinis* [46], and *Cutaneotrichosporon curvatus* [47] was 5.5. At acidic pH 3.0 and 4.0, the yeast biomass, lipid yield, and lipid content were decreased significantly because low acidic pH may lessen the metabolic functioning of the enzymes [35].

Oleaginous microorganisms require different incubation periods for maximum biomass production and lipid accumulation. The maximum production of biomass (17.95 g/L) and the highest lipid content (45.18%) were obtained after 168 h. Longer incubation periods resulted in a significant decrease in the biomass yield as well as lipid content. This decrease could be attributed to the utilization of storage lipids as an energy source after the depletion of fermentable sugars in the medium, as reported in the previous studies [48]. Jiru et al. [39] observed that SY89 gave maximum biomass (16.08 ± 0.78 g/L) at the end of 168-h incubation. However, this yeast gave maximum lipid yield (7.65 ± 0.77 g/L) and lipid content (51.17 ± 1.72%) after a growth period of 144 h.

Oleaginous yeasts are proposed to require a substantial supply of oxygen for energy [30, 49]. One factor that determines the amount of oxygen in the medium is the rate of agitation. By increasing the agitation rate, the dissolved oxygen in the medium increases, increasing the growth and lipid content [50]. Maximum biomass of 17.71 g/L, lipid yield of 8.15 g/L, and lipid content of 46.02% were observed by *R*. *diobovata* when the agitation rate was held at 200 rpm. Other researchers also studied the optimum agitation rate for different oleaginous yeasts. For example, the optimum agitation rate for the production of lipid by *R*. *kratochvilovae* was 225 rpm [39] and *R*. *glutinis* was 180 rpm [51]. Moreover, the maximum dry cell biomass and lipid yield were obtained at 200 rpm by *C*. *curvatus NRRLY-1511* [47]*.*

It has been found that light irradiation can induce the growth and development of varieties of organisms including fungi and their secondary metabolites such as carotenoids [52]. Mohamed and Valadon [53] reported that carotenogenesis in *Verticillium agaricinum* was under strict photo control but total lipid production was present in fairly large amounts in the dark. In the present study, the lipid yield was increased in dark conditions. Similar results for *Rhodotorula* strains were also reported in some studies. For example, Kong et al. [54] informed that light treatment did not improve the lipids content of *R*. *mucilaginosa*. Radiation could enhance the growth of *R*. *mucilaginosa*, but the rapid cell growth rate reversely inhibited the accumulation of lipid. Pham et al. [55] indicated that there was no observable difference in lipid content, with slight changes in fatty acid composition after light incubation in the case *of Rhodosporodium toruloids*.

Cultivation in a medium with excess carbon and a limited level of nitrogen has a significant effect on cell growth and lipid accumulation in oleaginous yeasts [56]. In the nitrogen-limited condition, the excess amount of carbon in the medium is used to produce lipid bodies by oleaginous yeasts. Huang et al. [57] reported that organic nitrogenous compounds are good for lipid accumulation, but not for cell growth; on the other hand, inorganic nitrogenous compounds are favorable for cell growth, but not for lipid accumulation. In the present study, corn steep liquor was proved to be the most suitable nitrogen source for lipid production, with 46.54% lipid content. According to other researchers, different nitrogen sources support different oleaginous yeasts either in combination or alone. For example, Zhu et al. [4] reported that urea supported maximum biomass, while peptone was the best nitrogen source for lipid production by *Geotrichum* (*syn*, *Trichosporon*). On the other hand, Kraisintu et al. [45] suggested that *Rhodotorula* (*syn*, *Rhodosporidium*) *toruloides* DMKU3-TK16 produced the highest biomass when the yeast was grown in a medium containing yeast extract and (NH_4_)_2_SO_4_ as nitrogen sources. Jiru et al. [39] stated that (NH_4_)_2_SO_4_ and yeast extract are the optimum nitrogen sources for SY89.

It has been shown that nitrogen is necessary for growth but the limited condition is also important for lipid production. Whereas, the present study showed that the use of molasses as substrate not only provided high lipid content (46.66%). Furthermore, there was no requirement to increase or decrease the culture temperature. Therefore, this process may make energy-saving possible. The experiments also demonstrated that a pH drop occurred during cell cultivation, especially between 24 and 96. After 168 h, the final pH of the medium was determined as 3.2. This decrease was probably due to the excretion of some organic acids. Similar results for *Rhodotorula* strains were also reported in some studies. For example, Calvente et al. [58] informed that the yeasts *R. glutinis* and *Rhodotorula rubra* produced rhodotorulic acid in the medium containing ammonium sulfate, thereby decreasing the culture pH. Cho et al. [59] demonstrated that when the extracellular polysaccharide production by *R*. *glutinis* was performed, the final pH of culture dropped below 2.0 (initial pH was 4.0) in the presence of ammonium salts (ammonium sulfate, ammonium chloride, ammonium nitrate). They presumed that the utilization of ammonium ions as a nitrogen source produced an expulsion of protons from the cells, causing the medium to become acidic.

When corn steep liquor was tested at the different concentrations, the maximum lipid content (46.68%) was observed at the CSL concentration of 1%. Applications above 1% resulted in a significant reduction in lipid yield and lipid content. This inhibitory effect can be explained by the fact that excessive nitrogen source or low C:N ratio limits lipid accumulation in oleaginous microorganisms including *Rhodotorula* strains [60]. In media with a high C/N ratio, enough carbon will remain in the medium and will be used for lipid accumulation during cell growth [61].

The fatty acid profiles of oleaginous yeasts depend on the species and the growth conditions. Environmental conditions, such as temperature, pH, substrate, C/N ratio, and oxygen influence the efficiency with which lipids are accumulated [62].

The lipids produced by *R*. *diobovata* were positive for the production of unsaturated fatty acids, showing resemblance to the composition of vegetable oils normally used for the production of biodiesel [62].

## Conclusions

Yeasts are promising microbial resources to produce various compounds, including lipids, which are of great interest to the biodiesel industry. In this regard, certain Egyptian habitats were evaluated for the isolation of lipid-producer wild yeasts, with the goal of producing microbial lipids from molasses as a raw material to accumulate fatty acids. The isolated *Rhodotoula diobovata* is capable of utilizing the molasses sucrose as a carbon source as well as tolerating high molasses concentrations with great potential for the accumulation of lipids, reaching concentrations above 45%. The optimization of cultivation conditions of the studied yeast strain gave maximal lipid production of 8.36 g/L lipid and cellular lipid content of 46.68% of dry biomass. The fatty acid profiles of the investigated strain revealed that its lipid content was similar to that of vegetable oil and consequently could be used for biodiesel production.

## Data Availability

All data generated or analyzed during this study are included in this article.

## Abbreviations

TAG: Triacylglycerol
YEPD: Yeast extract peptone dextrose
SCO: Single cell oil
FAME: Fatty acid methyl ester
NCBI: National Center For Biotechnology Information
BLAST: Basic Local Alignment Search Tool
CSL: Corn steep liquor
